# PORTRAYAL OF A CENTENARIAN: EXPLORING ACTOR INSIGHTS ON AGING AND LONGEVITY

**DOI:** 10.1093/geroni/igz038.1123

**Published:** 2019-11-08

**Authors:** Alex J Bishop, Tanya Finchum, Julie Pearson-Littlethunder

**Affiliations:** 1 Oklahoma State University, Stillwater, Oklahoma, United States; 2 Oklahoma Oral History Research Program Oklahoma State University, Stillwater, Oklahoma, United States

## Abstract

Data for this study originated the Oklahoma 100 Year Life Project. A total N = 7 volunteer actors (n = 5 women; n = 2 men) were recruited to participant as actors to play the role of centenarians in a living history play based on oral historical narratives. Actors participated in preliminary (prior to play performance) and post (after play performance) focus group sessions. Focus group questions addressed four key ideas: (1) Perceptions of aging and human longevity; (2) Loss and decline in aging; (3) Narrative storytelling and; (4) Personal life goal(s). Qualitative content analysis was performed to assess general thematic evidence stemming from actor perspectives. Four predominant themes emerged suggestive of adaptation in actor perceptions about human aging and longevity. These themes included: (1.) Purpose seeking (e.g. "It was interesting to see how much you can get out of life."); (2.) Age-embodiment (e.g., “I really wanted to focus on my facial movements and the way I talked”); (3.) Creative curiosity (e.g., “It takes a little bit of creativity. . . someone who has curiosity”); and (4.) Self-actualization (e.g., “You just think it’s not going to happen to me. . . and then you’re like. . . I am probably going to live to 100 now.”). Results have implications relative to how oral historical narratives can be used to enhance the personification of aging on stage, as well as demystify personal myths about longevity. Applications for use within educational and community settings for theatrical performances will be shared.

